# Preventive Effect of Daiokanzoto (TJ-84) on 5-Fluorouracil-Induced Human Gingival Cell Death through the Inhibition of Reactive Oxygen Species Production

**DOI:** 10.1371/journal.pone.0112689

**Published:** 2014-11-12

**Authors:** Kaya Yoshida, Masami Yoshioka, Hirohiko Okamura, Satomi Moriyama, Kazuyoshi Kawazoe, Daniel Grenier, Daisuke Hinode

**Affiliations:** 1 Department of Oral Healthcare Education, Institute of Health Biosciences, University of Tokushima Graduate School, Tokushima, Japan; 2 Department of Oral Health Science and Social Welfare, Institute of Health Biosciences, University of Tokushima Graduate School, Tokushima, Japan; 3 Department of Histology and Oral Histology, Institute of Health Biosciences, University of Tokushima Graduate School, Tokushima, Japan; 4 Department of Hygiene and Oral Health Science, Institute of Health Biosciences, University of Tokushima Graduate School, Tokushima, Japan; 5 Department of Clinical Pharmacy, Institute of Health Biosciences, University of Tokushima Graduate School, Tokushima, Japan; 6 Oral Ecology Research Group, Faculty of Dentistry, Laval University, Quebec City, QC, Canada; University of California Merced, United States of America

## Abstract

Daiokanzoto (TJ-84) is a traditional Japanese herbal medicine (Kampo formulation). While many Kampo formulations have been reported to regulate inflammation and immune responses in oral mucosa, there is no evidence to show that TJ-84 has beneficial effects on oral mucositis, a disease resulting from increased cell death induced by chemotherapeutic agents such as 5-fluorouracil (5-FU). In order to develop effective new therapeutic strategies for treating oral mucositis, we investigated (i) the mechanisms by which 5-FU induces the death of human gingival cells and (ii) the effects of TJ-84 on biological events induced by 5-FU. 5-FU-induced lactate dehydrogenase (LDH) release and pore formation in gingival cells (Sa3 cell line) resulted in cell death. Incubating the cells with 5-FU increased the expression of nucleotide-binding domain and leucine-rich repeat containing PYD-3 (NLRP3) and caspase-1. The cleavage of caspase-1 was observed in 5-FU-treated cells, which was followed by an increased secretion of interleukin (IL)-1β. The inhibition of the NLRP3 pathway slightly decreased the effects of 5-FU on cell viability and LDH release, suggesting that NLRP3 may be in part involved in 5-FU-induced cell death. TJ-84 decreased 5-FU-induced LDH release and cell death and also significantly inhibited the depolarization of mitochondria and the up-regulation of 5-FU-induced reactive oxygen species (ROS) and nitric oxide (NO) production. The transcriptional factor, nuclear factor-κB (NF-κB) was not involved in the 5-FU-induced cell death in Sa3 cells. In conclusion, we provide evidence suggesting that the increase of ROS production in mitochondria, rather than NLRP3 activation, was considered to be associated with the cell death induced by 5-FU. The results also suggested that TJ-84 may attenuate 5-FU-induced cell death through the inhibition of mitochondrial ROS production.

## Introduction

Kampo formulations, which are traditional Japanese herbal medicines composed of crude herb extracts, have been prescribed in Japan for a wide variety of diseases for over 1500 years [1]. However, little research has been conducted on their potential beneficial effects on oral health. In a previous study, we investigated the effects of 27 Kampo formulations on the growth and virulence properties of *Porphyromonas gingivalis* (*P. gingivalis*), which is a major pathogen of chronic periodontitis and showed that Kampo formulations containing Rhubarb Rhizome (Daio), including Daiokanzoto (TJ-84), can decrease the growth of *P. gingivalis* and its adherence to oral epithelial cells, suggesting that they may have potential for preventing periodontal diseases [2]. Moreover, *in* *vitro* evidence has shown that some Kampo formulations can decrease inflammation and bacterial infections of oral mucosa. For example, Shosaikoto and Orento decrease the production of the inflammatory mediator prostaglandin E_2_ by lipopolysaccharide (LPS)-treated human gingival fibroblasts [3], [4]. Shosaikoto also increases the gene expression of antimicrobial peptides such as calprotectin by human oral epithelial cells [5]. Lastly, Rokumigan has been reported to reduce IL-6 secretion by LPS-stimulated gingival epithelial cells and fibroblasts and to promote wound healing in a fibroblast model [6]. These results indicate that Kampo formulations may be promising new drugs for the prevention and treatment of oral mucosal diseases in which an inflammatory host response is involved.

5-fluorouracil (5-FU) is a widely used chemotherapeutic agent in the treatment of cancers. While 5-FU displays beneficial antitumor effects by inhibiting DNA synthesis [7], it also induces a high rate of oral mucositis (20–50%) in patients receiving multicycle chemotherapy [8]. Oral mucositis results from increased inflammation and the death of oral mucosal cells (epithelial cells and fibroblasts), and has specific symptoms such as erythema, bleeding, ulcer formation, and localized oral superinfections. The development of oral mucositis causes severe pain, which in turn makes it difficult to eat and drink, leading to malnutrition. Furthermore, the loss of the integrity of the oral mucosal epithelium favors the destruction of the mucosal barrier and increases the risk of local infections by oral pathogenic microorganisms such as *Candida albicans*, herpes simplex virus (HSV), and Gram-negative bacilli [9]. It has also been reported that the high prevalence of local infections associated with oral mucositis may increase the risk of systemic bacterial infections [10]. The prevention or treatment of oral mucositis may thus play a significant role in improving the quality of life and clinical outcomes of patients with cancer. While various strategies to prevent or treat oral mucositis have been evaluated, there is currently no effective therapeutic modality for this disease [11].

The reactive oxygen species (ROS) are involved in multiple biological processes leading to oral mucositis by both direct and indirect mechanisms [12], [13]. More specifically, 5-FU-induced ROS cause oxidative stress that damages DNA and proteins in epithelial cells, leading to cell death and ulcer formation, a characteristic of oral mucositis. The factors which are released from injured tissues affect the initiation and development of chemotherapy-induced mucositis [14]. 5-FU-induced ROS production also causes indirect effects through the activation of a number of signal transduction pathways that regulate transcriptional factors such as nuclear factor-κB (NF-κB). NF-κB modulates the expression of many genes that play critical roles in inflammatory cytokine secretion. The inflammatory response induced by these cytokines contributes to a loss of mucosal integrity and the progression of oral mucositis.

5-FU also induces and activates inflammasomes, multi-protein complexes formed by the intracellular nucleotide-binding domain and leucine-rich repeat containing PYD (NLRP) family, as well as apoptosis-associated speck-like protein containing a CARD (ASC) [15], [16]. NLRP3 inflammasomes have been extensively studied as they have been associated with many diseases, including type 2 diabetes mellitus [17], [18], cancer [19], Alzheimer’s disease [20], and atherosclerosis [21]. When NLRP3 inflammasomes recognize pathogenic microorganisms and danger signals they are activated and cleave pro-caspase-1. Caspase-1 possesses enzymatic activity and can induce inflammatory cell death called pyroptosis. Activated caspase-1 also leads to the cleavage and secretion of the biologically active form of interleukin (IL)-1β, an inflammatory cytokine. This regulation of cell death and cytokine production by NLRP3 inflammasomes may play important roles in immune and inflammatory responses [22]. It has recently been suggested that ROS, which are produced in mitochondria in response to various stimuli, trigger the activation of inflammasomes [23]. For example, ATP-mediated ROS increases the activation of caspase-1 and IL-1β and IL-18 secretion through by phosphatidylinositol 3-kinase (PI3 K) pathway in macrophages [24]. Asbestos and silica can induce ROS generation by NADPH oxidase, which leads to NLRP3 inflammasome activation [25]. It has also been proposed that ROS generation resulted from mitochondria dysfunction are required to activate NLRP3 inflammasomes [26]–[28]. Indeed, an NLRP3 gene mutation has been shown to induce autoinflammatory diseases such as cryopyrin-associated periodic syndrome (CAPS) and to alter the basal redox state of monocytes of patients with CAPS [29].

The findings described above suggest that NLRP3 inflammasomes are involved in the pathogenesis of 5-FU-associated oral mucositis through ROS production, although its role in oral mucositis has not yet been examined. In the present study, we looked at whether the NLRP3 inflammasome pathway is involved in 5-FU-induced Sa3 cell death with the ultimate goal of developing effective strategies to prevent or treat oral mucositis. We also looked at whether Kampo formulation Daiokanzoto (TJ-84) has a beneficial effect on oral mucositis by affecting the biological processes induced by 5-FU such as cell death, mitochondrial dysfunction, ROS generation, and NLRP3 inflammasome activation.

## Results

### 5-FU-induced Sa3 cell death

**Figure 1:**
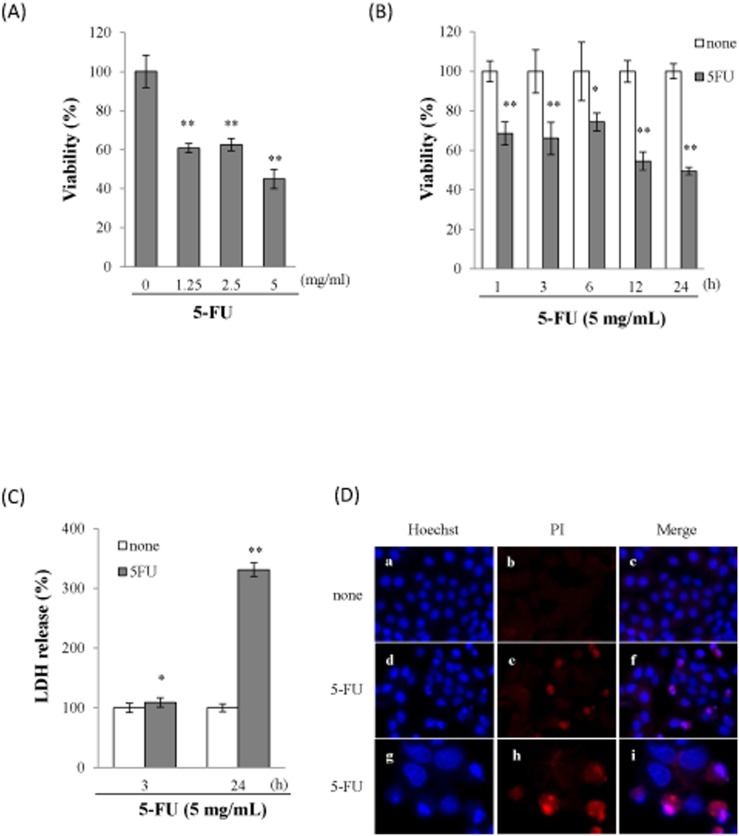
5-FU induced Sa3 cell death. (A), Viability of Sa3 cells incubated with various concentrations of 5-FU for 24 h. Values are means ± S.E.M. (n = 8). **p<0.01 compared to untreated cells. (B), Time course of cell viability of Sa3 cells incubated with 5 mg/mL of 5-FU. Values are means ± S.E.M. (n = 8). *p<0.05, **p<0.01 compared to control cells. (C), LDH levels in culture media after 3-h and 24-h incubations with 5-FU. Data shown are percentages with respect to control cells at each time point. Values are means ± S.E.M. (n = 8). *p<0.05, **p<0.01 compared to Sa3 cells incubated without 5-FU. (D), Micrographs of Sa3 cells incubated without (a–c) or with (d–i) 5-FU for 24**h. Hoechst 33342- (a, d, g) and PI-stained cells (b, e, h) and merged images (c, f, i) are shown. Micrographs of cells incubated with 5-FU for 24 h at high magnification (×1,000) (g–i).

To investigate the involvement of 5-FU in cell death, Sa3 cells were incubated with different concentrations of 5-FU for 24 h prior to measuring cell viability. The incubation of the cells with increasing concentrations of 5-FU (1.25–5 mg/mL) resulted in decrease in cell viability over a 24-h period (Fig. 1A). A time-course study showed that there was a significant decrease in cell viability from 1 to 24 h following the exposure of the Sa3 cells to 5 mg/mL of 5-FU (Fig. 1B). In order to determine whether 5-FU-induced cell death was related to cell lysis, the release of cytosolic LDH into the extracellular environment was quantified. A significant release of LDH into the supernatant was observed within 3 h and increased up to 24 h following the incubation with 5-FU (Fig. 1C). Given that 5-FU-induced LDH release suggested that 5-FU led to cell lysis, we investigated pore formation in response to 5-FU by assessing the uptake of propidium iodide (PI) and Hoechst 33342. As shown in Figure 1D, all the cells were stained with the membrane-permeant dye Hoechst 33342 (Fig. 1D a, d, g) whereas only cells with membrane pores allowed the membrane-impermeant dye PI to diffuse into the cells (Fig. 1D e, h). The influx of PI was observed 24 h after the incubation with 5-FU (Fig. 1D e, h) whereas PI did not diffuse into cells that had not been incubated with 5-FU (Fig. 1D b). These results indicated that 5-FU induced pore formation in Sa3 cells.

### Involvement of NLRP3 inflammasomes in 5-FU-induced cell death

**Figure 2:**
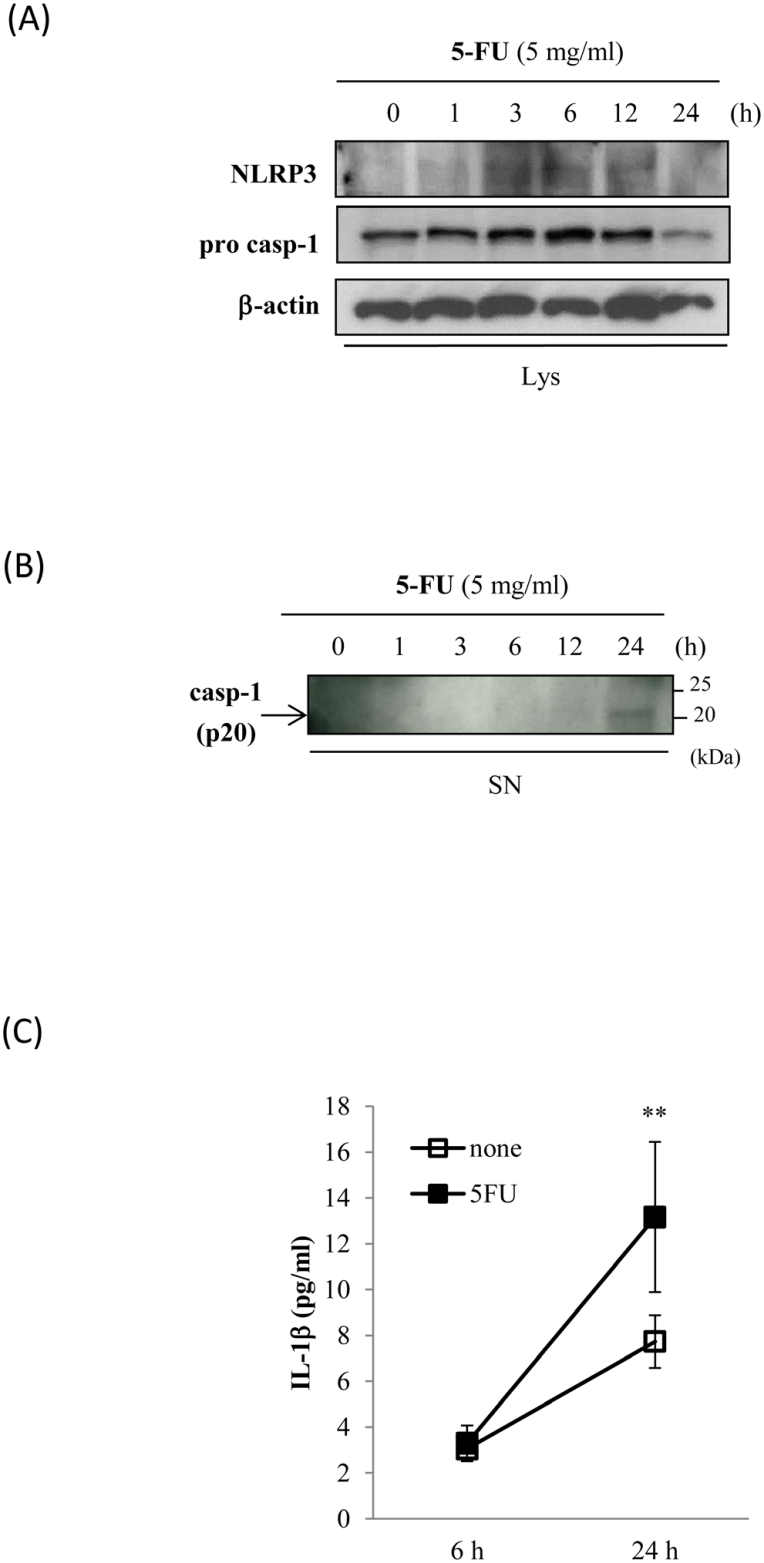
5-FU-activated inflammasome pathway. Sa3 cells were incubated with 5 mg/mL of 5-FU for 0 to 24 h. (A), Western blot analysis of the expression of NLRP3 and the precursor of caspase-1 (pro-casp-1) in cell lysates. (B), Western blot analysis of cleaved caspase-1 (p20) in supernatants. Arrow indicates p20-specific bands. (C), ELISA assay of IL-1β in supernatants of Sa3 cells incubated without (open box) or with (closed box) 5 mg/mL of 5-FU for 6 h and 24 h. Values are means ± S.E.M. (n = 4). **p<0.01 compared to Sa3 cells incubated without 5-FU.

**Figure 3:**
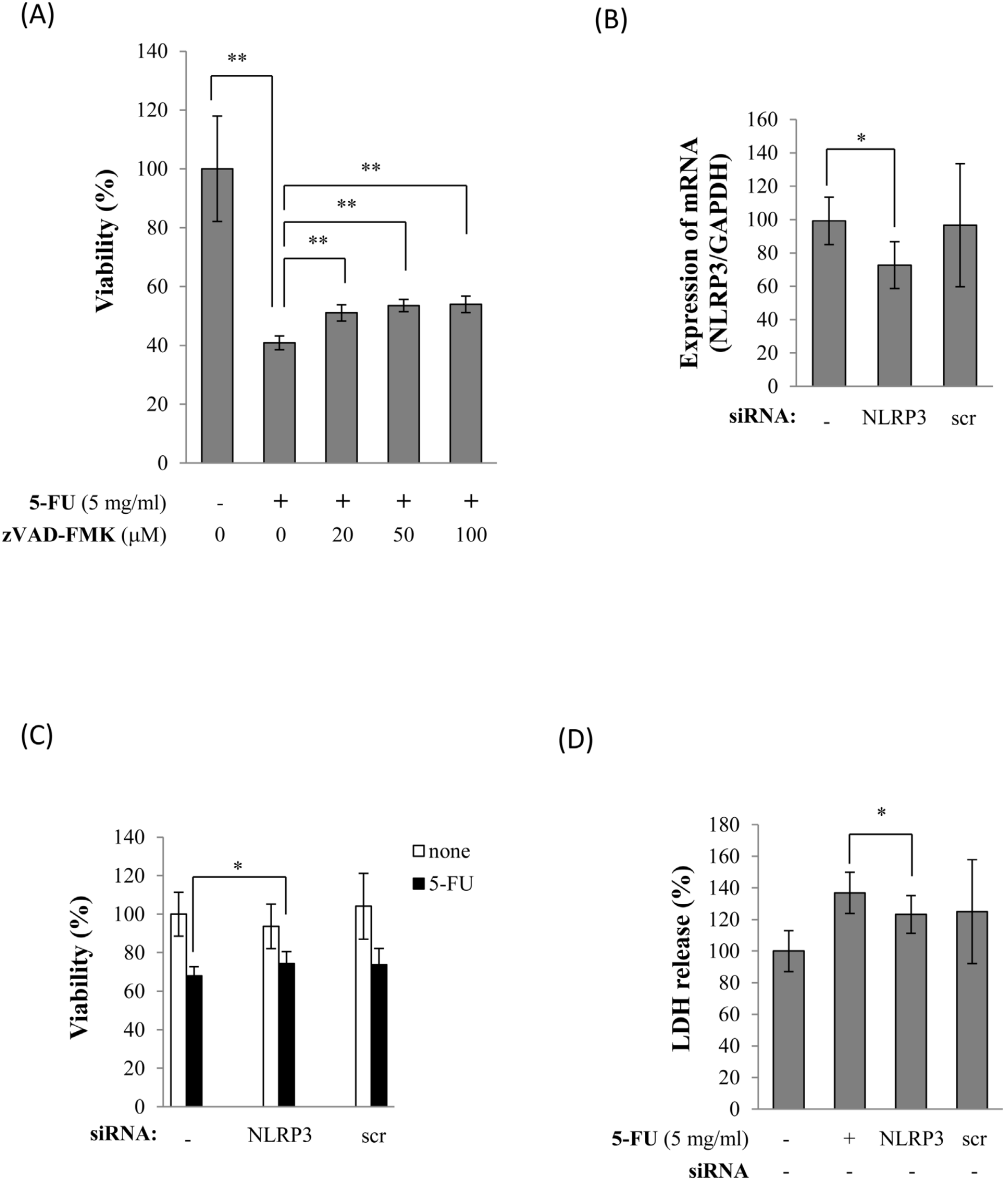
Inhibition of NLRP3 inflammasomes decreased 5-FU-induced cell death. (A), Cell viability of Sa3 cells incubated with 5 mg/mL of 5-FU for 24 h after a 30-min pre-incubation with caspase inhibitor at each concentration. Values are means ± S.E.M. (n = 4). **p<0.01 compared to the control group. (B), Expression of NLRP3 mRNA in Sa3 cells treated with NLRP3 siRNA (NLRP3) or scrambled oligo (scr). Values are means ± S.E.M. (n = 4). *p<0.05 compared to cells without oligo (–). (C), Cell viability of cells transduced without (–) or with NLRP3 siRNA (NLRP3) or control oligo (scr) following an incubation with (closed bar) or without (open bar) 5 mg/mL of 5-FU for 3 h. Data are given as percentages compared to the group that was not incubated with 5-FU. Values are means ± S.E.M. (n = 6). *p<0.05 compared to the control group. (D), Effect of transfection with NLRP3 siRNA (NLRP3) or scrambled oligo (scr) on 5-FU-induced LDH release. The LDH levels in the supernatants are given as percentages of cells not incubated with 5-FU and not transduced with siRNA. Values are means ± S.E.M. (n = 8). *p<0.05 compared to the control cells.

We hypothesized that if NLRP3 inflammasomes are activated in response to 5-FU, caspase-1 would be cleaved to the p20 subunit, which in turn would produce and release the mature form of IL-1β. To verify this hypothesis, we used Western blot analyses to determine whether 5-FU affects the expression of NLRP3 and the caspase-1 p20 subunit in Sa3 cells. The incubation with 5-FU increased NLRP3 protein expression between 3 and 12 h after the initiation of the incubation. Pro-caspase-1 levels increased at 6 h after the initiation of the incubation with 5-FU (Fig. 2A). Caspase-1 was cleaved and secreted into the supernatant at 24 h after the initiation of the incubation (Fig. 2B). IL-1β secretion into the supernatant was quantified by ELISA following a 6-h or 24-h incubation of the cells with 5 mg/mL 5-FU. Increased secretion of IL-1β at 24 h post-5-FU incubation was observed compared to cells that had not been incubated with 5-FU (Fig. 2C). Given that 5-FU activated the inflammasome pathway, we then investigated whether the NLRP3 inflammasome pathway regulates 5-FU-induced Sa3 cell death. Sa3 cells were pre-incubated for 30 min with 0 to 100 µM carbobenzoxy-valyl-alanyl-aspartyl-[*O*-methyl]-fluoromethylketone (zVAD-FMK), a caspase inhibitor that binds to the catalytic site of the enzyme. They were then incubated with 5 mg/mL of 5-FU for 24 h after which cell viability was measured. Figure 3A shows that pre-incubating the cells with zVAD-FMK attenuated the decrease in cell viability induced by 5-FU. We then knocked down NLRP3 using siRNA and determined whether the reduction in NLRP3 expression affects cell viability and LDH release in response to 5-FU. The expression of NLRP3 mRNA was suppressed in siRNA-treated cells but not in control cells. The scrambled oligonucleotide did not affect NLRP3 mRNA expression (Fig. 3B). Cell viability was not altered by the NLRP3 knock-down itself, while 5-FU-suppressed cell viability (68.08±4.62%) was slightly higher in NLRP3 knock-down cells (74.26±6.28%, p = 0.042) (Fig. 3C). The siRNA knock-down of NLRP3 decreased LDH release (123.18±11.87%, p = 0.045), the scrambled oligonucleotide had no effect (124.99±32.90%, p = 0.945), while 5-FU increased LDH release (136.87±12.99%) (Fig. 3D).

### Preventive effects of TJ-84 on 5-FU-induced cell death

**Figure 4:**
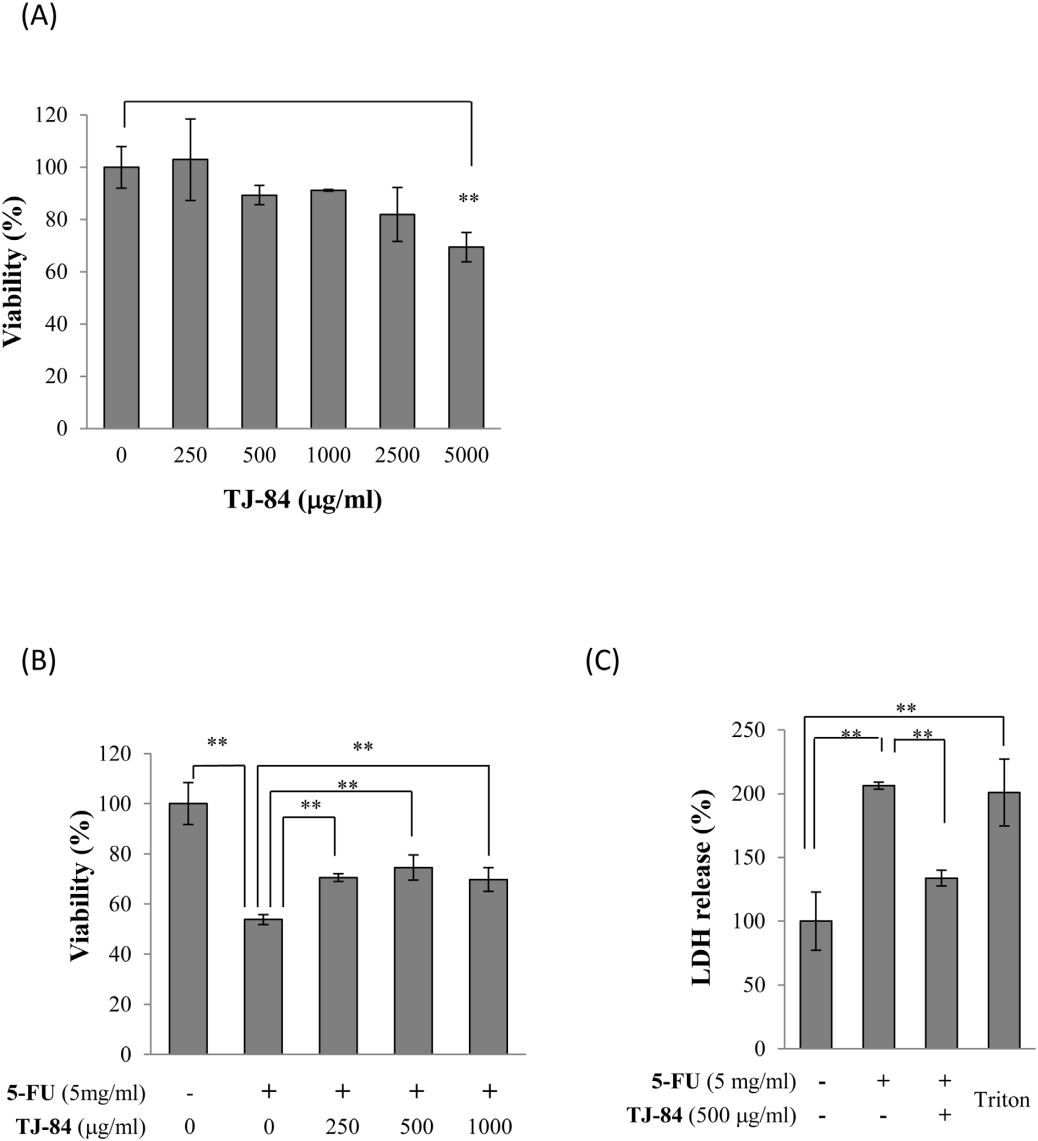
TJ-84 reduced 5-FU-induced cell death. (A), Cytotoxicity of TJ-84. Sa3 cells were incubated with various concentrations of TJ-84 for 24 h, and cell viability was then measured using WST-8 kits. Values are means ± S.E.M. (n = 3). **p<0.01 compared to control cells that had not been incubated with TJ-84. (B), Viability of cells incubated with various concentrations of TJ-84 for 1 h and then incubated with 5-FU for 24 h. Results are expressed as percentages with respect to control cells that had not been incubated with TJ-84 and 5-FU. Values are means ± S.E.M. (n = 4). **p<0.01 compared to control cells. (C), The effect of TJ-84 on LDH release from cells incubated with 5-FU for 24 h was assessed using WST-8 kits. The supernatant of Sa3 cells incubated with 0.1% Triton-X for 5 min was used as a positive control (Triton). The LDH levels in the supernatants are expressed as percentages with respect to cells that had not been incubated with 5-FU. Values are means ± S.E.M. (n = 4). *p<0.05 compared to the control cells.

To determine whether TJ-84 can prevent 5-FU-induced cell death, we first evaluated the cytotoxic effect of TJ-84 on Sa3 cells. Sa3 cells were incubated with TJ-84 at concentrations up to 5000 µg/mL for 24 h, and cell viability was then assessed using a WST-8 assay. While up to 2500 µg/mL of TJ-84 had no toxic effect on Sa3 cells, cell viability decreased significantly at 5000 µg/mL (Fig. 4A). We then incubated Sa3 cells with TJ-84 at concentrations ranging from 0 to 1000 µg/mL for 1 h, incubated them with 5-FU for 24 h, and then assessed cell viability. Concentrations of TJ-84 ranging from 250 µg/mL up to 1000 µg/mL attenuated 5-FU-suppressed cell viability (Fig. 4B). To determine whether TJ-84 attenuates the secretion of LDH induced by 5-FU, Sa3 cells were pre-incubated with 500 mg/mL of TJ-84 for 1 h. They were then incubated with 5 mg/mL of 5-FU for 24 h, and LDH levels in the supernatant were measured. Since Triton-X permeabilizes the cell membrane, which leads to the release of the cytosolic contents into the medium, we used cells incubated with 0.1% Triton-X for 5 min at room temperature as a positive control for LDH release. As expected, the incubation of the cells with 0.1% Triton-X increased the release of LDH. The 24-h incubation with 5 mg/mL 5-FU increased LDH release, while the pre-incubation with TJ-84 significantly attenuated LDH release (Fig. 4C).

### TJ-84 reduces mitochondrial ROS production

**Figure 5:**
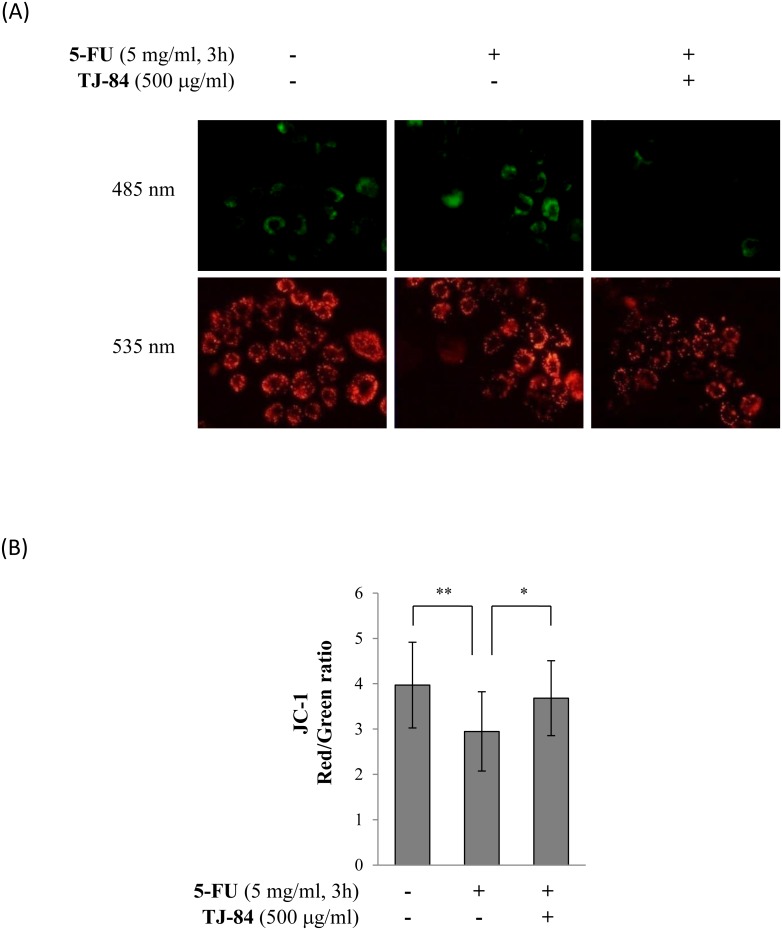
TJ-84 attenuated 5-FU-induced mitochondrial depolarization. (A), Sa3 cells were incubated with or without 5 mg/mL of 5-FU for 3 h following a 1-h pre-incubation with 500 µg/mL of TJ-84. JC-1 (1 µg/mL) was then loaded for 30 min. JC-1 aggregates (red) and monomers (green) were detected by fluorescence microscopy. (B), The fluorescence intensity per cell was calculated using ImageJ. The calculation of the red/green ratio is shown on the graph. Values are means ± S.E.M. (n = 20, 16, 14). **p<0.01, *p<0.05 compared to the control cells.

We examined the effects of 5-FU and TJ-84 on ROS production by mitochondria to investigate the molecular mechanisms by which TJ-84 attenuates the death of 5-FU-incubated Sa3 cells. Since mitochondrial depolarization occurs in the early stages of cell death, we first determined whether 5-FU and TJ-84 modify the membrane potential of mitochondria using JC-1. Monomer JC-1 is excited by green fluorescence (488 nm) and selectively accumulates in the mitochondrial matrix where it forms red fluorescence (568) JC-1 aggregates. Mitochondrial depolarization can thus be visualized as a shift in fluorescence from red to green. Cells were incubated with 5 mg/mL of 5-FU for 3 h followed by 500 µg/mL of TJ-84 for 1 h and then with 1 µg/mL of JC-1 for 30 min. The cells were observed by fluorescence microscopy. The accumulation of JC-1 aggregates (red fluorescence) decreased in 5-FU-incubated cells, whereas the accumulation of JC-1 monomers (green fluorescence) increased compared to cells that had not been incubated with 5-FU (Fig. 5A). These results suggested that 5-FU may decrease the accumulation of JC-1 in the mitochondrial membrane by inducing its depolarization. The 5-FU-induced reduction in membrane depolarization was recovered by a pre-incubation with TJ-84 (Fig. 5A). Fluorescence intensity was quantified in each group using NIH ImageJ analysis software, and the red/green fluorescence intensity ratio was calculated. Consistent with the results shown in Figure 5A, the red/green fluorescence intensity ratio was lower in Sa3 cells incubated with 5-FU for 3 h (2.948±0.876, p = 0.002) than in cells that had not been incubated with 5-FU (3.970±0.947). The red/green ratio suppressed by 5-FU wacorrecteds recovered by a pre-incubation with TJ-84 (3.682±0.826, p = 0.026) (Fig. 5B). These results suggested that TJ-84 inhibits the 5-FU-induced depolarization of the mitochondrial membrane of Sa3 cells.

**Figure 6:**
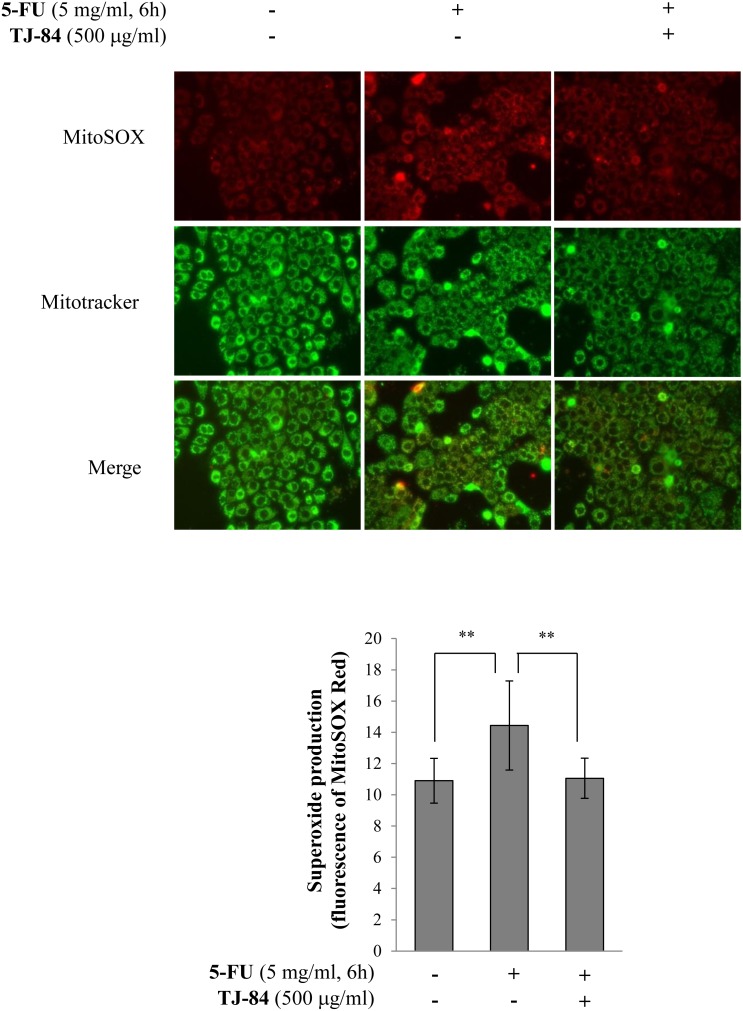
TJ-84 suppressed 5-FU-induced ROS production in mitochondria. (A), Sa3 cells were incubated with or without 5 mg/mL of 5-FU for 6 h following a 1-h pre-incubation with 500 µg/mL of TJ-84. MitoSOX Red (5 µM) and 100 µM Mitotracker Green were then loaded for 30 min. ROS (red) and mitochondria (green) were detected by fluorescence microscopy. (B), The red fluorescence intensity per cell was calculated using ImageJ and is shown on the graph. Values are means ± S.E.M. (n = 63, 71, 75). **p<0.01 compared to the control cells.

Mitochondrial impairment results in the production of ROS. We thus examined the effects of 5-FU and TJ-84 on the generation of mitochondria-specific ROS. We assessed mitochondria-specific ROS levels using MitoSOX Red, which selectively detects mitochondria-derived O·^ ¯^_2_ but not other ROS such as hydrogen peroxide (H_2_O_2_), hydroxyl radicals (OH·), and reactive nitrogen species. The localization of mitochondria was also assessed using Mitotracker Green. Red fluorescence detected by MitoSOX Red was higher in Sa3 cells incubated with 5 mg/ml 5-FU for 6 h than in cells that had not been incubated with 5-FU (Fig. 6A). The 5-FU-induced increase in red fluorescence was suppressed by a 1-h pre-incubation with TJ-84 (Fig. 6A). These results were quantified using NIH ImageJ analysis software. The incubation with 5-FU increased the intensity of red fluorescence after 3 h (14.435±2.852, p<0.01) compared to cells that had not been incubated with 5-FU (10.901±1.429), while TJ-84 significantly inhibited the effect of 5-FU (11.058±1.284, p<0.01) (Fig. 6B). These results indicated that TJ-84 decreases the generation of mitochondria-derived-O_2_·that is up-regulated by 5-FU.

### The effects of NF-κB and nitric oxide on 5-FU-induced cell death

**Figure 7:**
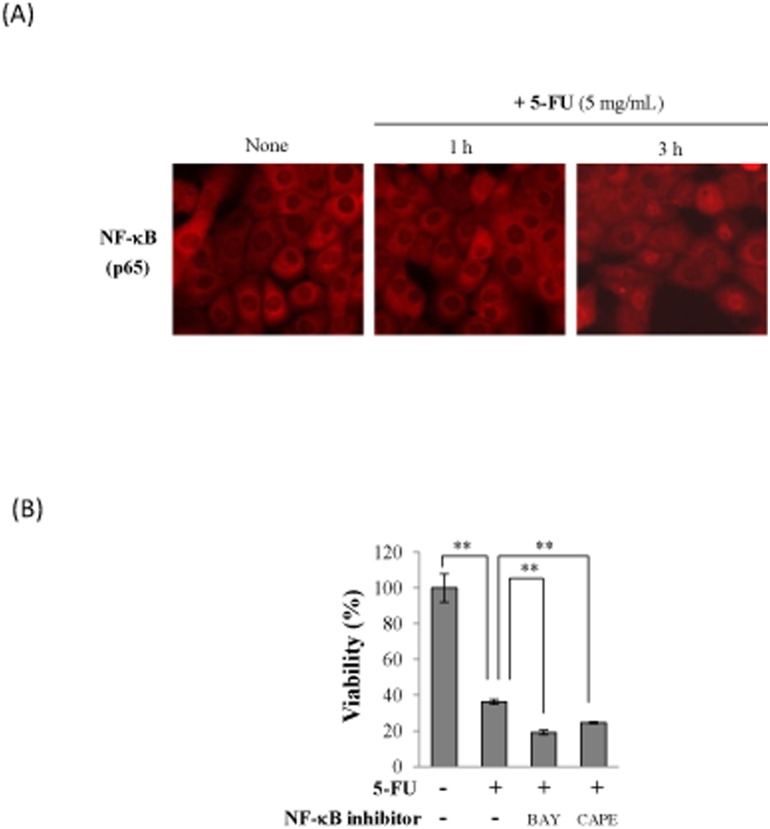
NF-κB did not attenuate 5-FU-induced cell death. (A), Sa3 cells were incubated with or without 5 mg/mL of 5-FU for 1 h and 3 h. The cells then stained with anti-p65 antibody and the localization of p65 was detected by florescence microscopy. (B), Cell viability of Sa3 cells incubated with 5 mg/mL of 5-FU for 24 h after a 30-min pre-incubation with NF-κB inhibitors, 5 µM BAY or 200 µM CAPE. Values are means ± S.E.M. (n = 4). **p<0.01 compared to the control group.

To investigate the further mechanisms of 5-FU-induced oral mucositis, we examined the effects of 5-FU on the transcriptional factor, NF-κB in Sa3 cells. Activated NF-κB translocates from the cytosol to the nucleus where it regulates gene expression. We thus determined whether 5-FU affects the localization of NF-κB in Sa3 cells by immunocytochemistry using an antibody directed against p65, a subunit of NF-κB. NF-κB translocated from the cytoplasm to the nucleus 3 h after the initiation of the 5-FU incubation (Fig. 7A), suggesting that 5-FU increased the active form of NF-κB. We next investigated the effect of NF-κB on 5-FU-induced cell death by a pre-incubation with BAY 11-7085 and caffeic acid phenethyl ester (CAPE), two inhibitors of NF-κB activation. The inhibition of NF-κB by BAY 11-7085 or CAPE did not allow the 5-FU-induced loss of Sa3 cell viability to be recovered (Fig. 7B).

**Figure 8:**
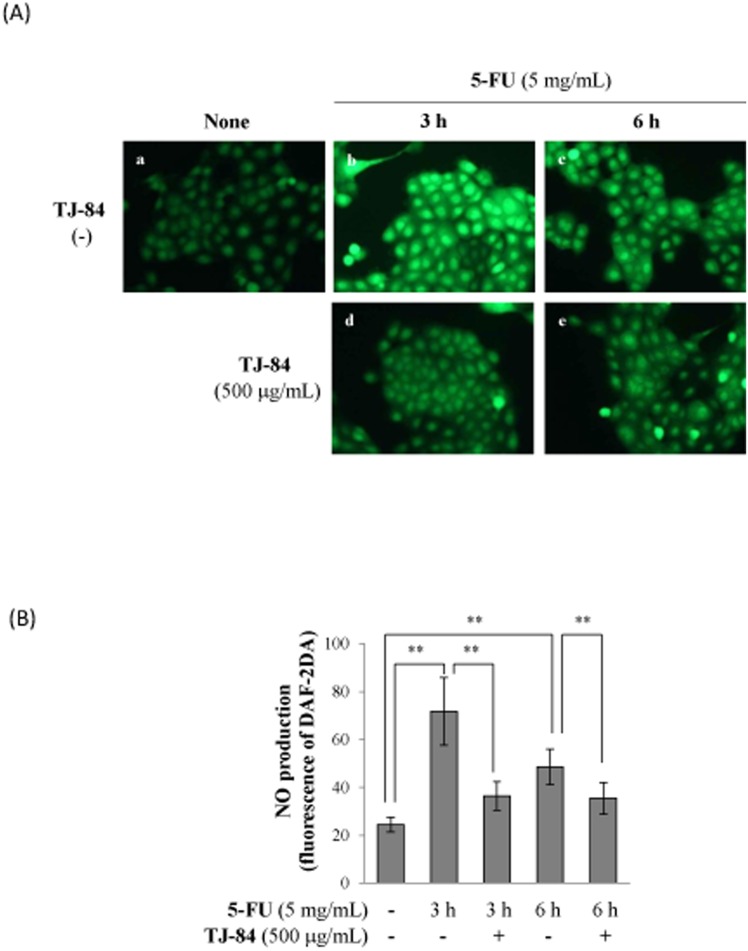
TJ-84 attenuated 5-FU-induced NO production. (A), Sa3 cells were incubated with or without 5 mg/mL of 5-FU for 3 h following a 1-h pre-incubation with 500 µg/mL of TJ-84. DAF-2DA was then loaded for 30 min. The production of NO (green) were detected by fluorescence microscopy. (B), The fluorescence intensity per cell was calculated using ImageJ. The calculation of the red/green ratio is shown on the graph. Values are means ± S.E.M. (n = 50). **p<0.01 compared to the control cells.

We next examined the effect of 5-FU on the production of nitric oxide (NO) by Sa3 cells. The production of NO was determined by DAF-2DA, a cell-permeable sensitive fluorescent indicator. After the incubation with DAF-2DA, cells were analyzed in fluorescence microscope. Green fluorescence detected by DAF-2DA was higher in Sa3 cells incubated with 5 mg/mL 5-FU for 3h (Fig. 8A, b) and 6 h (Fig. 8A, c) than in cells that had not been incubated with 5-FU (Fig. 8A, a). The 5-FU-induced increase in green fluorescence was suppressed by a 1-h pre-incubation with TJ-84 (Fig. 8A, d, e). These results were quantified using NIH ImageJ software. The incubation with 5-FU increased the intensity of green fluorescence after 3 h (71.69±14.11, p<0.01) and 6 h (48.53±7.40, p<0.01) compared to cells that incubated without 5-FU (24.47±3.05), while TJ-84 significantly inhibited the effect of 5-FU at both 3 h (36.46±5.95, p<0.01) and 6 h (35.46±6.64, p<0.01) (Fig. 8B).

## Discussion

We report two main findings: (i) 5-FU-activated NLRP3 inflammasome induces gingival cell death at low NLRP3 levels, and (ii) TJ-84 suppresses 5-FU-induced mitochondrial ROS production and, as a result, 5-FU-induced cell death.

Inflammasomes, including NLRP3 and NLRP1, are expressed at high levels in hematopoietic cells such as granulocytes, dendritic cells, and B and T cells. However, Kummer *et al*. reported that NLRP3 inflammasomes, but not NLRP1 inflammasomes, are also expressed in the epithelia of the oropharynx and esophagus, suggesting that NLRP3 inflammasomes in the digestive tract may allow the sensing of invading pathogens [30]. It has also been reported that NLRP3 inflammasomes are expressed in fibroblasts and epithelial cells of the oral mucosa and that their expression is regulated by oral bacterial infections [31]–[33]. Consistent with these reports, we detected NLRP3 in Sa3 cells, which are derived from human gingiva epithelial cells, and observed that NLRP3 was up-regulated by 5-FU (Fig. 2A).

Pyroptosis triggered by caspase-1 activation through the inflammasome pathway is programmed cell death associated with inflammation and is different from apoptosis. In our experimental models, 5-FU increased the cleavage of caspase-1, which led to the release of IL-1β by Sa3 cells (Fig. 2). Based on these results, 5-FU-induced cell death appeared to be related to pyroptosis, while, in the past, 5-FU has been reported to induce apoptosis, resulting in the progression of oral mucositis. To confirm that 5-FU-induced cell death was indeed pyroptosis, we analyzed the features of pyroptotic cell death, including cell lysis and pore formation, in Sa3 cells. Pyroptosis results in cell lysis and the release of cytosolic contents such as LDH [34]. In contrast, cytosolic contents are not released during apoptosis because they are contained in vesicles called apoptotic bodies, which are shed by blebbing [35]. As shown in Figure 1C, the release of LDH into the supernatant increased significantly between 3 and 24 h post-5-FU incubation. Pore formation was assessed using the membrane impermeable dye PI since active caspase-1 induces ion-permeable pores in the plasma membrane in cells dying by pyroptosis [36]. PI was incorporated into Sa3 cells within 24 h of the 5-FU incubation (Fig. 1D). These results suggested that Sa3 cell death induced by 5-FU is related to pyroptosis. Moreover, 5-FU-reduced cell viability was slightly increased by inhibiting the NLRP3 inflammasome pathway using a caspase inhibitor or NLRP3 siRNA (Fig. 3A, C). 5-FU-induced release of LDH was also suppressed by NLRP3 siRNA (Figure 3D). These results suggested that the pyroptotic cell death induced by 5-FU is regulated, at least in part, by the caspase-1/NLRP3 inflammasome pathway.

It was recently reported that ROS-activated inflammasome increases intestinal mucositis in mice treated with chemotherapeutic agent, irinotecan [37]. Administration of IL-1 receptor antagonist to the mouse model also reduces 5-FU-induced intestinal mucositis [38]. These observations indicate that inflammasome plays critical roles in chemotherapy-induced intestinal mucositis. In present study, 5-FU slightly increased the secretion of IL-1β (Fig. 2C), while inhibiting NLRP3 did not entirely recover the 5-FU-induced decrease in cell viability (Fig. 3C), suggesting that other mechanisms may be involved in 5-FU-induced cell death in addition to the NLRP3/caspase-1 pathway. 5-FU also significantly increased mitochondrial ROS production (Figs. 5 and 6). It is thus possible that other factors induced by ROS are involved in 5-FU-induced cell death. We verified two possible candidates: the NF-κB-regulated apoptosis pathway and NO production.

ROS can act as a modulator of signal transduction following the activation of transcriptional factors such as NF-κB, AP-1, and p53. In chemotherapy-induced oral mucositis, NF-κB is the most important transcriptional factor [39] and can cause apoptosis by increasing the expression of BCL-2 family genes. As shown in Figure 7, NF-κB was translocated to nucleus by 5-FU treatment, however, the treatment with NF-κB inhibitors did not recover the 5-FU-induced loss of Sa3 cell viability. These results indicated that NF-κB is activated by 5-FU but is not involved in 5-FU-induced cell death in our experimental model.

In contrast, our results indicated that NO, which was regulated by 5-FU, may be involved in 5-FU-induced Sa3 cell death (Figure 8). However, the role of NO is controversial since it can contribute both positively and negatively to cell death [40], [41]. The unpaired electron (NO·) can react with a superoxide radical (O·^ ¯^_2_) to form the powerful oxidant peroxynitrite (ONOO·^−^), which is thought to induce apoptosis via multiple mechanisms, including the induction of p53 and ER stresses, the release of cytochrome c by mitochondrial transition, and the activation of p38 or other MAP kinases [42]. Our results with MitoSOX Red showed that 5-FU increased the production of mitochondria-derived superoxide radicals and that TJ-84 inhibited this production (Fig. 6), suggesting that peroxynitrite, which is produced by a reaction between the superoxide radical and ROS, may contribute to 5-FU-induced Sa3 cell death. On the other hand, low levels of NO are thought to inhibit cell death [43], [44], while NO negatively regulates NLRP3 inflammasomes via the S-nitrosylation of NLRP3 [45]. It remains unclear whether 5-FU-induced NO can form peroxynitrite and mediate the activation of pro-apoptotic pathways. Further studies will be required to examine the effect of NO on 5-FU-induced cell death and its relationship with ROS.

Our results clearly showed that TJ-84 attenuates the 5-FU-induced decrease in Sa3 cell viability, indicating that Kampo formulation TJ −84 shows potential as a therapeutic agent for the treatment of 5-FU-induced oral mucositis. A number of naturally occurring compounds in plants, including Kampo formulations, have been investigated for their ability to reduce the severity of 5-FU-induced mucositis. For example, Iberogast, a herbal formula composed of nine extracts, possesses anti-inflammatory properties and has been shown to partially improve the histopathological features of mucositis in the small intestines of rats injected intraperitoneally with 5-FU [46]. Moreover, topically applying Kampo formulation Hangeshashinto to the oral mucosa decreases the symptoms of oral mucositis in patients with advanced colorectal cancer undergoing chemotherapy [47].

Additional properties associated to TJ-84 or its ingredients may also contribute to maintaining healthy oral mucosa. Unpublished data obtained in our laboratory showed that TJ-84 possesses an anti-inflammatory activity resulting in a decreased secretion of inflammatory cytokines by lipopolysaccharide-stimulated gingival epithelial cells and fibroblasts. Moreover, both a licorice extract and emodin, an anthraquinone derivative from rhubarb, have been shown to possess wound healing properties. In a preliminary study, Das *et al.* reported that the use of a mouthwash containing a deglycerinized licorice extract for two weeks tends to provide pain relief and accelerate the healing of aphthous ulcers [48]. More recently, Tang *et al.* showed in a rat model that emodin promotes wound healing through transforming growth factor-β1 (TGF-β1)/Smad signaling pathway [49]. These reports support our results and suggest that Kampo formulations can improve oral mucositis.

Kampo formulations have been used to treat a number of diseases, and their beneficial effects have been widely acknowledged. However, the mechanisms by which Kampo formulations produce their effects are not well understood. In the present study, the treatment of TJ-84 suppressed 5-FU-induced mitochondrial ROS production (Fig. 6) and NO production (Fig. 8). It has been reported that Kampo formulation Inchinkoto possesses antioxidant properties that act via a nuclear factor-E2 (Nrf2)-dependent mechanism [50], and that Inchinkoto suppresses Fas-mediated apoptosis in the liver [51]. Based on our results and these reports, it is possible that TJ-84 may decrease 5-FU-induced cell death by decreasing mitochondrial-associated oxidative stresses in Sa3 cells.

**Figure 9:**
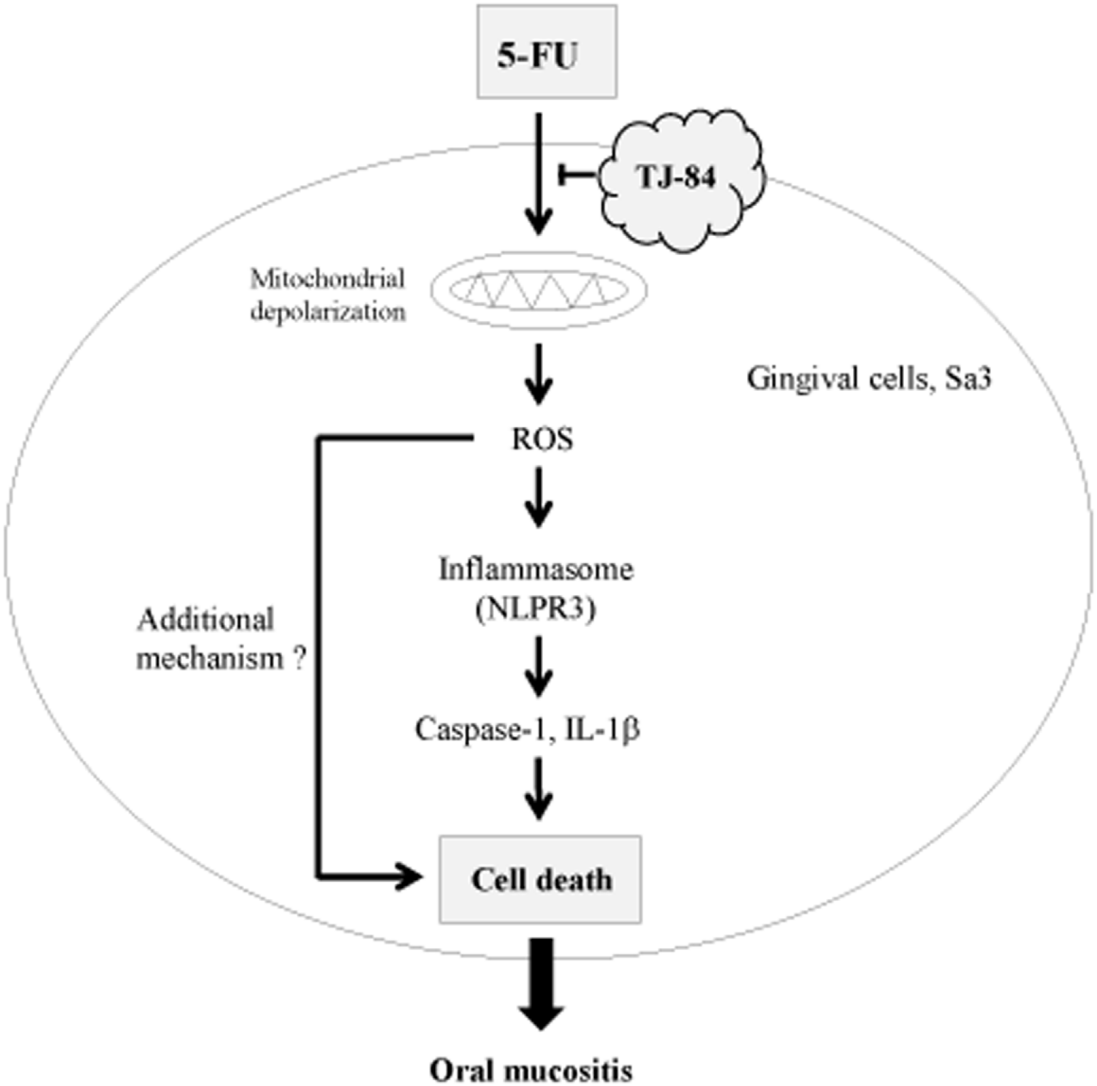
Hypothetical model of how TJ-84 decreases 5-FU-induced Sa3 cell death. TJ-84 decreased 5-FU-induced cell death by inhibiting ROS production. See text for details.

In conclusion, we showed that 5-FU-induced Sa3 cell death involves ROS and the NLRP3 inflammasome pathway (Fig. 9). 5-FU caused mitochondrial depolarization and an up-regulation of ROS production, which triggered the activation of NLRP3 inflammasomes and caspase-1, resulting in an increase in cell death. In addition to the NLRP3 inflammasome pathway, another unknown mechanism appears to participate in 5-FU-induced cell death. Kampo formulation TJ-84 may prevent the loss of cell viability by inhibiting the effect of 5-FU on mitochondrial ROS production. Our findings point to a new mechanism by which 5-FU induces cell death in oral mucositis and suggest that TJ-84 may be useful in treating 5-FU-induced oral mucositis.

## Materials and Methods

### Drugs

Daiokanzoto (TJ-84) was obtained from Tsumura & Co (Tokyo, Japan). TJ-84 is manufactured as a powdered extract obtained from spray drying a decoction of 2 medicinal plants: 4.0 g of Rhubarb Rhizome (Daio) and 2.0 g of Glycyrrhiza Root (Kanzo). TJ-84 was dissolved in hot water homogeneously at a concentration of 500 mg/mL and used for experiments. 5-fluorouracil (5-FU injection 250 Kyowa) was purchased from Kyowa Hakko Kirin (Tokyo, Japan).

### Cell cultures

Sa3 OSCC cells were kindly provided by the RIKEN BioResource Center through the National BioResource Project of MEXT (Ministry of Education, Culture, Sports, Science & Technology, Tokyo, Japan). The Sa3 cells were plated in plastic dishes at a density of 10×10^4^ cells/mL and were cultured in DMEM supplemented with 10% fetal bovine serum (FBS) at 37°C in a humidified 5% CO_2_/95% air atmosphere. After reaching 70−80% confluence, the cells were used for the experiments.

### Cell viability

Cell viability was assessed using WST-8 Cell Counting Kit-8 assays (Dojindo Laboratories, Kumamoto, Japan, cat. no 347-07621). Briefly, Sa3 cells were plated at a density of 1×10^4^ cells per well in 96-well plates. After the cells had been incubated with 5-FU for the indicated periods, 10 µL of kit reagent was added to the wells. Following a 30-min incubation, cell viability was assessed using a ELISA plate reader.

### LDH release

Sa3 cells were incubated with 5-FU for the indicated periods, and aliquots of culture medium were then collected to measure extracellular LDH activity. As a positive control for LDH release, 0.1% Triton-X was added into the medium and incubated for 5 min at room temperature.LDH activity was monitored using LDH cytotoxicity assay kits (Cayman Chemical Company, Ann Arbor, MI, USA, cat no 10008882) according to the manufacturer’s protocol.

### Propidium iodide (PI) and Hoechst 33342 staining

Sa3 cells were seeded onto coverslips and were incubated with or without 5-FU for the indicated periods. Following the 5-FU incubation, the cells were incubated with 1 µg/mL of PI or 1 µg/mL of Hoechst 33342 for 15 min. Adhered cells were fixed, mounted, and examined under a microscope. Images were acquired using an ECLIPSE Ti-U microscope and NIS-Elements software (Nikon, Tokyo, Japan).

### IL-1β release

Sa3 cells were incubated with 5-FU for the indicated periods, and aliquots of culture medium were then collected. IL-1β was measured with a Quantikine ELISA (R&D systems, Minneapolis, MN, USA) according to manufacture instructions (cat no. DLB50).

### siRNA

An RNA duplex targeting the 5′-GUUGCAAGAUCUCUCAGCA-3′ sequence of human NLRP3 (NM_004894.5) was synthesized and transfected into Sa3 cells using Lipofectamine 2000 (Invitrogen, Carlsbad, CA, USA, cat no 11668027). A scrambled oligonucleotide, which was designed to have no homology to known gene sequences, was transfected into Sa3 cells as a negative control. After 24 h, the transfected cells were incubated with or without 5-FU and were used for the experiments.

### Real-time PCR

Total RNA was isolated from Sa3 cells using ISOGEN (Nippon Gene, Tokyo, Japan, cat no 347-07621), followed by phenol extraction and ethanol precipitation. The cDNA was synthesized using Prime Script RT reagent kits (Takara Bio, Kyoto, Japan, cat no RR037A). Real-time PCR was performed with a 7300 Real-Time PCR system (Applied Biosystems, Carlsbad, CA, USA) using SYBR Premix Ex Taq (Takara Bio, cat no RR820A). The primer sequences were as follows: human GAPDH (NM_002046): forward, 5′-GCACCGTCAAGGCTGAGAAC-3′, reverse, 5′-TGGTGAAGACGCCAGTGGA-3′; human NLRP3 (NM_004894.5): forward, 5′-AAGCACCTGTTGTGCAATCTGAAG-3′, reverse, 5′-GGGAATGGCTGGTGCTCAATAC-3′.

### SDS-PAGE and Western blot analyses

Sa3 cells were washed with PBS and were scraped into TN lysis buffer (50 mM Tris [pH 8.0], 150 mM NaCl, 0.1% NP-40) supplemented with protease inhibitors (4 µg/mL of aprotinin, 1 µg/mL of leupeptin, 0.2 mM phenylmethylsulfonyl fluoride (PMSF)). The surpernatants were collected and aliquots of them (3 mL) were concentrated to 250 µL using 10 kDa Amicon Ultra centrifuge tubes (Merck KGaA, Darmstadt, Gemany, cat no UFC801024). The protein extracts were immunoblotted using a previously published protocol [52]. Anti-caspase-1 p20 antibody was from Enzo Life Science (Farmingdale, NY, USA, cat no ALX-210-804). Anti-pro-caspase-1 and anti-β-actin antibodies were from Santa Cruz Biotechnology (Santa Cruz, CA, USA, cat no sc-515, sc-47778). Anti-NLRP3 antibody (Cryo-2) was from Adipogen (Liestal, Switzerland, cat no AG-20B-0014).

### Mitochondrial depolarization

Sa3 cells were incubated with 1 µg/mL of 5, 5′, 6, 6′-tetrachloro-1, 1′, 3, 3′-tetraethyl-benzimidazol-carbocyanin iodide (JC-1) (Molecular Probe, Invitrogen, Milan, Italy, cat no) for 30 min and were detected at 590/610 nm (excitation/emission) for JC-1 aggregates and 485/535 nm (excitation/emission) for JC-1 monomers by fluorescence microscopy. The fluorescence intensity per cell was quantified using NIH ImageJ analysis software, and the ratio of red/green was calculated.

### Mitochondrial ROS production

To detect surperoxide in mitochondria, MitoSOX Red (Molecular Probe, Invitrogen, cat no M36008) and MitoTracker Green FM (Molecular Probe, Invitrogen, cat no M-7514) were used according to the manufacturers’ instructions. Briefly, cells were incubated with 5-FU or/and TJ-84 for the indicated periods and were then loaded with MitoSOX Red (5 µM) and MitoTracker Green FM (100 µM) in balanced salt solution [BSS; 135 mM NaCl, 5.6 mM KCl, 1.2 mM MgSO_4_, 2.2 mM CaCl_2_, 10 mM glucose, and 20 mM [4-(2-hydroxyethyl)-1-piperazine ethanesulfonic acid (HEPES)/NaOH, pH 7.4] for 20 min. The cells were rinsed with BSS, and the locations of ROS and mitochondria were observed using a fluorescence microscope (ECLIPSE Ti-U, Nikon). The red fluorescence intensity per cell from images obtained by fluorescence microscopy was quantified by NIH ImageJ software.

### Immunocytochemistry and NF-κB inhibitors

Sa3 cells were cultured on sterile 18-mm round coverslips. After reaching 70−80% confluence, the cells were treated with 5 mg/mL 5-FU for 1 or 3 h. For immunocytochemistry, the cells were fixed with 3.0% formalin for 30 min and then permeabilized with 0.1% Triton X-100 in PBS for 2 min at 4°C. After blocking of nonspecific binding sites, cells were incubated with anti-NF-κB antibody (sc-372) (Santa cruz) diluted 1∶200 in 4% BSA in PBS overnight at 4°C, followed by Alexa 546-conjugated anti-rabbit IgG (Molecular Probes, Eugene, OR, cat no A11010), diluted 1∶500 in 4% BSA in PBS for 60 min at ambient temperature. The samples were mounted and examined under a microscope equipped with epifluorescence illumination (ECLIPSE Ti-U, Nikon). To exmamine the effect of NF-κB on 5-FU-suppressed cellular viability, Sa3 cells incubated with NF-κB inhibitors, 5 µM BAY 11-7085 (Enzo Life Sciences, cat no EI-279) and 200 µM Caffeic acid phenethyl ester (CAPE) (Calbiochem, San Diego, CA, cat no 211200) for 1 h. Then cells were treated with 5 mg/mL 5-FU for 24 h and cellular viability was mesured by WST-8 assay.

### NO production

Sa3 cells were pre-treated with 500 mg/mL TJ-84 for 1 h before treatment with 5 mg/mL 5-FU for 3 or 6 h. Then the cells were incubated with the 10% FBS DMEM containing the mixture of 10 µM diaminofluorescein-2 diacetate (DAF-2 DA, Sekisui medical, Kyoto, Japan, cat no 423727) and pluronic F-127 (50/50%, v/v) for 1 h, and then fixed with 10% formalin. The levels of NO were assessed by fluorescence microscopy at 488 nm (ECLIPSE Ti-U, Nikon). The fluorescence intensity was quantified by Image J software.

### Statistical analysis

All data are expressed as means ± S.E.M. A minimum of three independent experiments were performed for each assay. The Student’s *t*-test was used for the statistical analyses.

## References

[1] Watanabe K, Matsuura K, Gao P, Hottenbacher L, Tokunaga H, et al.. (2011) Traditional Japanese Kampo medicine: Clinical research between modernity and traditional medicine–The state of research and methodological suggestions for the future. Evid Based Complement Alternat Med 2011, doi: 10.1093/ecam/neq067. 10.1093/ecam/neq067 PMC3114407 21687585

[2] Liao J, Zhao L, Yoshioka M, Hinode D, Grenier D (2013) Effects of Japanese traditional herbal medicines (Kampo) on growth and virulence properties of *Porphyromonas gingivalis* and viability of oral epithelial cells. Pharm Biol 51: 1538–1544. 23987742 10.3109/13880209.2013.801995

[3] Ara T, Maeda Y, Fujinami Y, Imamura Y, Hattori T, et al.. (2008) Preventive effects of a Kampo medicine, Shosaikoto, on inflammatory responses in LPS-treated human gingival fibroblasts. Biol Pharm Bull 3:, 1141–1144. 10.1248/bpb.31.1141 18520044

[4] Ara T, Honjo K, Fujinami Y, Hattori T, Imamura Y, et al (2010) Preventive effects of a Kampo medicine, Orento on inflammatory responses in lipopolysaccharide treated human gingival fibroblasts. Biol Pharm Bull 33: 611–616. 20410594 10.1248/bpb.33.611

[5] Hiroshima Y, Bando M, Kataoka M, Shinohara Y, Herzberg MC, et al (2009) Shosaikoto increases calprotectin expression in human oral epithelial cells. J Periodont Res 2010 45: 79–86. 10.1111/j.1600-0765.2009.01203.x 19602113

[6] Liao J, Jabrane A, Zhao L, Yoshioka M, Hinode D, et al.. (2014) The Kampo medicine Rokumigan possesses antibiofilm, anti-inflammatory, and wound healing properties. Biomed Res Int 2014, Article ID 436206. 10.1155/2014/436206 PMC4022067 24877093

[7] Fata F, Ron IG, Kemeny N, O’Reilly E, Klimstra D, et al (1999) 5-fluorouracil-induced small bowel toxicity in patients with colorectal carcinoma. Cancer 86: 1129–1134. 10506695 10.1002/(sici)1097-0142(19991001)86:7<1129::aid-cncr5>3.0.co;2-4

[8] Peterson DE, Bensadoun RJ, Roila F (2011) Management of oral and gastrointestinal mucositis: ESMO Clinical Practice Guidelines. Ann Oncol 22: vi78–vi84. 21908510 10.1093/annonc/mdr391 PMC3662500

[9] Dreizen S, Bodey GP, Valdivieso M (1983) Chemotherapy-associated oral infections in adults with solid tumors. Oral Surg Oral Med Oral Pathol 55: 113–120. 6572860 10.1016/0030-4220(83)90164-0

[10] Elting LS, Rubenstein EB, Rolston KV, Bodey GP (1997) Outcomes of bacteremia in patients with cancer and neutropenia: observations from two decades of epidemiological and clinical trials. Clin Infect Dis 25: 247–259. 9332520 10.1086/514550

[11] Saadeh CE, Pharm D (2005) Chemotherapy-and radiotherapy-induced oral mucositis: review of preventive strategies and treatment. Pharmacotherapy 25: 540–554. 15977916 10.1592/phco.25.4.540.61035

[12] Sonis ST (2004) The pathobiology of mucositis. Nat Rev 4: 277–284. 10.1038/nrc1318 15057287

[13] Yoshino F, Yoshida A, Nakajima A, Wada-Takahashi S, Takahashi S, et al (2013) Alteration of the redox state with reactive oxygen species for 5-fluorouracil-induced oral mucositis in hamsters. PLoS ONE 8: e82834. 24376587 10.1371/journal.pone.0082834 PMC3869731

[14] Sonis ST (2010) New thoughts on the initiation of mucositis. Oral Dis 16: 597–600. 20846150 10.1111/j.1601-0825.2010.01681.x

[15] Masters SL, Gerlic M, Metcalf D, Preston S, Pellegrini M, et al (2012) NLRP1 inflammasome activation induces pyroptosis of hematopoietic progenitor cells. Immunity 37: 1009–1023. 23219391 10.1016/j.immuni.2012.08.027 PMC4275304

[16] Bruchard M, Mignot G, Derangère V, Chalmin F, Chevriaux A, et al (2013) Chemotherapy-triggered cathepsin B release in myeloid-derived suppressor cells activates the Nlrp3 inflammasome and promotes tumor growth. Nat Med 19: 57–64. 23202296 10.1038/nm.2999

[17] Schroder K, Zhou R, Tschopp J (2010) The NLRP3 inflammasome: A sensor for metabolic danger? Science 327: 296–300. 20075245 10.1126/science.1184003

[18] Lee BC, Lee J (2014) Cellular and molecular players in adipose tissue inflammation in the development of obesity-induced insulin resistance. Biochim Biophys Acta 1842: 446–462. 23707515 10.1016/j.bbadis.2013.05.017 PMC3800253

[19] Okamoto M, Liu W, Luo Y, Tanaka A, Cai X, et al (2010) Constitutively active inflammasome in human melanoma cells mediating autoinflammation via caspase-1 processing and secretion of interleukin-1β. J Biol Chem 285: 6477–6488. 20038581 10.1074/jbc.M109.064907 PMC2825443

[20] Halle A, Hornung V, Petzold GC, Stewart CR, Monks BG, et al (2008) The NALP3 inflammasome is involved in the innate immune response to amyloid-β. Nat immunol 9: 857–865. 18604209 10.1038/ni.1636 PMC3101478

[21] Duewell P, Kono H, Rayner KJ, Sirois CM, Vladimer G, et al (2010) NLRP3 inflammasomes are required for atherogenesis and activated by cholesterol crystals. Nature 464: 1357–1362. 20428172 10.1038/nature08938 PMC2946640

[22] Ouyang X, Ghani A, Mehal WZ (2013) Inflammasome biology in fibrogenesis. Biochimica et Biophysica Acta 183: 979–988. 10.1016/j.bbadis.2013.03.020 23562491

[23] Lawlor KE, Vince JE (2014) Ambiguities in NLRP3 inflammasome regulation: Is there a role for mitochondria? Biochim Biophys Acta 1840: 1433–1440. 23994495 10.1016/j.bbagen.2013.08.014

[24] Cruz CM, Rinna A, Forman HJ, Ventura AL, Persechini PM, et al (2007) ATP activates a reactive oxygen species-dependent oxidative stress response and secretion of proinflammatory cytokines in macrophages. J Biol Chem 282: 2871–2879. 17132626 10.1074/jbc.M608083200 PMC2693903

[25] Dostert C, Pétrilli V, Bruggen RV, Steele C, Mossman BT, et al (2008) Innate immune activation through Nalp3 inflammasome sensing of asbestos and silica. Science 320: 674–677. 18403674 10.1126/science.1156995 PMC2396588

[26] Nakahira K, Haspel JA, Rathinam VAK, Lee SJ, Dolinay T, et al (2011) Autophagy proteins regulate innate immune responses by inhibiting the release of mitochondrial DNA mediated by the NALP3 inflammasome. Nat immunol 8: 222–231. 10.1038/ni.1980 PMC3079381 21151103

[27] Zhou R, Yazdi AS, Menu P, Tschopp J (2011) A role for mitochondria in NLRP3 inflammasome activation. Nature 469: 221–225. 21124315 10.1038/nature09663

[28] Jabaut J, Ather JL, Taracanova A, Poynter ME, Ckless K (2013) Mitochondria-targeted drugs enhance Nlrp3 inflammasome-dependent IL-1β secretion in association with alterations in cellular redox and energy status. Free Rad Biol Med 60: 233–245. 23376234 10.1016/j.freeradbiomed.2013.01.025 PMC3705582

[29] Tassi S, Carta S, Delfino L, Caorsi R, Martini A, et al (2010) Altered redox state of monocytes from cryopyrin-associated periodic syndromes causes accelerated IL-1beta secretion. Proc Natl Acad Sci U S A 107: 9789–9794. 20445104 10.1073/pnas.1000779107 PMC2906851

[30] Kummer JA, Broekhuizen R, Everett H, Agostini L, Kuijk L, et al (2007) Inflammasome components NALP 1 and 3 show distinct but separate expression profiles in human tissue suggesting a site-specific role in the inflammatory response. J Histochem Cytochem 55: 443–452. 17164409 10.1369/jhc.6A7101.2006

[31] Belibasakis GN, Guggenheim B, Bostanci N (2013) Down-regulation of NLRP3 inflammasome in gingival fibroblasts by subgingival biofilms: involvement of *Porphyromonas gingivalis* . Innate Immun 19: 3–9. 22522430 10.1177/1753425912444767

[32] Bostanci N, Meier A, Guggenheim B, Belibasakis GN (2011) Regulation of NLRP3 and AIM2 inflammasome gene expression levels in gingival fibroblasts by oral biofilms. Cell Immunol 270: 88–93. 21550598 10.1016/j.cellimm.2011.04.002

[33] Yilmaz O, Sater AA, Yao L, Koutouzis T, Pettengill M, et al (2010) ATP-dependent activation of an inflammasome in primary gingival epithelial cells infected by *Porphyromonas gingivalis* . Cell Microbiol 12: 188–198. 19811501 10.1111/j.1462-5822.2009.01390.x PMC2807919

[34] Duprez L, Wirawan E, Berghe TV, Vandenabeele P (2009) Major cell death pathways at a glance. Microbes Infect 11: 1050–1062. 19733681 10.1016/j.micinf.2009.08.013

[35] Majno G, Joris I (1995) Apoptosis, oncosis, and necrosis. An overview of cell death. Am J Pathol 146: 3–15. 7856735 PMC1870771

[36] Fink SL, Cookson BT (2006) Caspase-1-dependent pore formation during pyroptosis leads to osmotic lysis of infected host macrophages. Cell Microbiol 8: 1812–1825. 16824040 10.1111/j.1462-5822.2006.00751.x

[37] Arifa RD, Madeira MF, de Paula TP, Lima RT, Tavares LD, et al (2014) Inflammasome activation is reactive oxygen species dependent and mediates irinotecan-induced mucositis through IL-1β and IL-18 in mice. Am J Pathol 184: 2023–2034. 24952429 10.1016/j.ajpath.2014.03.012

[38] Wu Z, Han X, Qin S, Zheng Q, Wang Z, et al (2010) Interleukin 1 receptor atagonist reduces lethality and intestinal toxicity of 5-fluorouracil in a mouse mucositis model. Biomed Pharmacother 64: 589–593. 20888173 10.1016/j.biopha.2010.06.006

[39] Sonis ST (2002) The biologic role for nuclear factor-kappaB in disease and its potential involvement in mucosal injury associated with anti-neoplastic therapy. Crit Rev Oral Biol Med 13: 380–389. 12393757 10.1177/154411130201300502

[40] Brüne B, von Knethen A, Sandau KB (1998) Nitric oxide and its role in apoptosis. Eur J Pharmacol 351: 261–272. 9721017 10.1016/s0014-2999(98)00274-x

[41] Brown GC (2010) Nitric oxide and neuronal death. Nitric Oxide 23: 153–165. 20547235 10.1016/j.niox.2010.06.001

[42] Brown GC, Borutaite V (2002) Nitric oxide inhibition of mitochondrial respiration and its role in cell death. Free Radic Biol Med 33: 1440–1450. 12446201 10.1016/s0891-5849(02)01112-7

[43] Takuma K, Phuagphong P, Lee E, Mori K, Baba A, et al (2001) Anti-apoptotic effect of cGMP in cultured astrocytes: inhibition by cGMP-dependent protein kinase of mitochondrial permeable transition pore. J Biol Chem 276: 48093–48099. 11677240 10.1074/jbc.M108622200

[44] Thomas DD, Ridnour LA, Isenberg JS, Flores-Santana W, Switzer CH, et al (2008) The chemical biology of nitric oxide: implications in cellular signaling. Free Rad Biol Med 45: 18–31. 18439435 10.1016/j.freeradbiomed.2008.03.020 PMC2572721

[45] Hernandez-Cuellar E, Tsuchiya K, Hara H, Fang R, Sakai S, et al (2012) Cutting edge: nitric oxide inhibits the NLRP3 inflammasome. J Immunol 189: 5113–5117. 23100513 10.4049/jimmunol.1202479

[46] Wright TH, Yazbeck R, Lymn KA, Whitford EJ, Cheah KY, et al (2009) The herbal extract, Iberogast, improves jejunal integrity in rats with 5-fluorouracil (5-FU)-induced mucositis. Cancer Biol Ther 8: 923–929. 19276679 10.4161/cbt.8.10.8146

[47] Kono T, Satomi M, Chisato N, Ebisawa Y, Suno M, et al (2010) Topical application of Hangeshashinto (TJ-14) in the treatment of chemotherapy-induced oral mucositis. World J Oncol 1: 232–235. 29147213 10.4021/wjon263w PMC5649748

[48] Das SK, Das V, Guati AK, Singh VP (1989) Deglycyrrhizinated liquorice in aphthous ulcers. J Assoc Physicians India 37: 647. 2632514

[49] Tang T, Yin Longwu, Yang Jing, Shan G (2007) Emodin, an antharquinone derivative from *Rheum officinale* Baill, enhances cutaneous wound healing in rats. Eur J Pharmacol 567: 177–185. 17540366 10.1016/j.ejphar.2007.02.033

[50] Okada K, Shoda J, Kano M, Suzuki S, Ohtake N, et al (2007) Inchinkoto, a herbal medicine, and its ingredients dually exert Mrp2/MRP2-mediated choleresis and Nrf2-mediated antioxidative action in rat livers. Am J Gastrointest Liver Physiol 292: G1450–G1463. 10.1152/ajpgi.00302.2006 17038627

[51] Yamamoto M, Miura N, Ohtake N, Amagaya S, Ishige A, et al (2000) Genipin, a metabolite derived from the herbal medicine Inchin-ko-to, and suppression of Fas-induced lethal liver apoptosis in mice. Gastroenterology 2000 118: 380–389. 10.1016/s0016-5085(00)70220-4 10648466

[52] Okamura H, Yang D, Yoshida K, Haneji T (2013) Protein phosphatase 2A Cα is involved in osteoclastogenesis by regulating RANKL and OPG expression in osteoblasts. FEBS Lett 587: 48–53. 23183242 10.1016/j.febslet.2012.10.041

